# Engineering Ion Affinity of Zr-MOF Hybrid PDMS Membranes for the Selective Separation of Na^+^/Ca^2+^

**DOI:** 10.3390/molecules29225297

**Published:** 2024-11-09

**Authors:** Ahmed S. Abou-Elyazed, Xiaolin Li, Jing Meng

**Affiliations:** 1 Institute of Intelligent Manufacturing Technology, Shenzhen Polytechnic University, Shenzhen 518055, China; ahmedabouelyazed@science.menofia.edu.eg; 2 Chemistry Department, Faculty of Science, Menoufia University, Shebin EL-Kom 32512, Egypt; 3 School of Civil Engineering, Nantong Institute of Technology, Nantong 226002, China

**Keywords:** hybrid membrane, PDMS, UiO-66, cation separation, diffusion coefficients

## Abstract

Ion-selective separation, especially Na^+^/Ca^2+^ separation, is of significant importance in the realms of biomimetic research and the fabrication of biomimetic devices, underscoring the pivotal role that sodium and calcium ions play in cellular metabolism. However, the analogous ionic radii and charge densities shared by sodium and calcium ions significantly impede their effective discrimination, presenting formidable challenges for the precise engineering of ion separation materials, such as separation membranes. In this study, a polydimethylsiloxane (PDMS) separation membrane hybridized with zirconium-based metal–organic frameworks (UiO-66, UiO-66-NO_2_ and UiO-66-NH_2_) was constructed. Through the meticulous design of the MOF functional groups, the material’s affinity for specific ions was modulated, thereby achieving efficient Na^+^/Ca^2+^ separation. Notably, the PDMS integrated with amino-modified Zr-MOF exhibited an efficacious selective separation of Na^+^ and Ca^2+^ ions. The interaction between the amino group of UiO-66-NH_2_ and Ca^2+^ gave rise to the observed superior selectivity toward Ca^2+^ cations and enhanced separation efficiencies of up to 64% compared to pristine PDMS for UiO-66-NH_2_-embedded membranes.

## 1. Introduction

Due to their capacity for efficient and economical separation procedures, membrane-based separation technologies have garnered considerable interest in recent times [1]. Among these technologies, ion exchange membranes (IEMs) have been identified as up-and-coming contenders for various applications, encompassing desalination, wastewater treatment, and resource recovery [2]. A specific focus is the separation of sodium and calcium cations, which are frequently encountered in industrial brine streams and provide notable difficulties for traditional separation techniques. Researchers have incorporated metal–organic frameworks (MOFs) into the membrane matrix to improve the selectivity and efficiency of ion exchange membranes [3,4]. Metal–organic frameworks (MOFs) are a crystalline material category consisting of metal ions or clusters aligned with organic ligands. These materials exhibit distinctive characteristics, including elevated porosity, adjustable pore diameters and selective adsorption abilities, rendering them very suitable for augmenting membrane efficacy [5,6].

Incorporating metal–organic frameworks (MOFs) into ion exchange membranes presents numerous benefits in sodium/calcium cation separation. The enhanced adsorption capacity of membranes is attributed to the increased surface area facilitated by the porous structure of metal–organic frameworks (MOFs) [7,8]. This process facilitates the effective elimination of specific cations while simultaneously reducing the adsorption of undesired ions. Furthermore, the adjustable characteristics of MOFs facilitate the personalization of pore dimensions and surface composition, hence permitting the targeted adsorption of particular cations. The selectivity of this platform can be customized to align with the specific demands of diverse separation processes, hence offering a flexible framework for a wide range of applications [9,10].

Several studies have demonstrated the effectiveness of MOF-embedded ion exchange membranes for ion separation. For example, a study by Li et al. investigated the incorporation of a calcium-selective MOF, MIL-101(Cr), into a commercially available ion exchange membrane [11]. The results showed enhanced calcium adsorption capacity and selectivity, leading to improved separation performance. Similarly, Zhang et al. reported the successful integration of a sodium-selective MOF, ZIF-8, into an ion exchange membrane, resulting in superior sodium removal efficiency [10]. Also, the integration of UiO-66 into ion exchange membranes has been employed to separate heavy metal ions, including cadmium and lead. The excellent adsorption capacity and selectivity toward target ions are facilitated by the porous structure of UiO-66 [12]. However, when it comes to sodium and calcium ion separation, several challenges and limitations need to be addressed to fully exploit the capabilities of these materials [10,13]. For example, the selective separation of sodium and calcium ions is challenging due to their similar ionic radii and charge densities. The small differences in their physical properties make it difficult for MOF-based membranes to achieve high selectivity [14]. The design and synthesis of MOFs with tailored pore sizes, functional groups and charge distribution are crucial to enhance selectivity for sodium and calcium ion separation. Furthermore, advancements in MOF synthesis and membrane fabrication techniques have facilitated the development of novel MOF-embedded ion exchange membranes with enhanced properties. For instance, nanocomposite membranes incorporating MOFs within a polymer matrix have shown improved stability and selectivity compared to conventional membranes. Additionally, the functionalization of MOFs with specific ligands has enabled further customization of membrane properties, enabling precise control over cation adsorption and separation [15].

Herein, the fabrication of Zr-MOF@PDMS membranes was carried out using three Zr-MOFs, namely UiO-66, UiO-66-NH_2_ and UiO-66-NO_2_, by the casting process. A systematic investigation was conducted to examine the morphology, structure and stability of the Zr-MOF@PDMS membranes. The study investigated the impact of the pore size and functional groups of Zr-MOFs on the Ca^2+^/Na^+^ separation property of Zr-MOF@PDMS membranes. Additionally, Zr-MOF loading at the constant thickness of Zr-MOF@PDMS membranes was optimized for Ca^2+^/Na^+^ transport and selectivity. Furthermore, the diffusion coefficients of Na^+^ and Ca^2+^ in the optimal Zr-MOF@PDMS membrane were conducted using a combination of the diffusion experiment and the electrochemical method. The UiO-66-NH_2_-0.05@PDMS membrane, which incorporates an amino group, exhibited remarkable stability and exceptional selectivity toward Ca^2+^ ions. This work compared the stability and efficiency of UiO-66 and its functionalized materials (UiO-66-NH_2_ and UiO-66-NO_2_) with suitable pore sizes for membrane-based Ca^2+^/Na^+^ separation and is expected to pave the way for developing a MOF-based membrane material for the general separation of sodium and calcium ions.

## 2. Results and Discussions

### 2.1. Zr-MOF@PDMS Membranes

**Figure 1:**
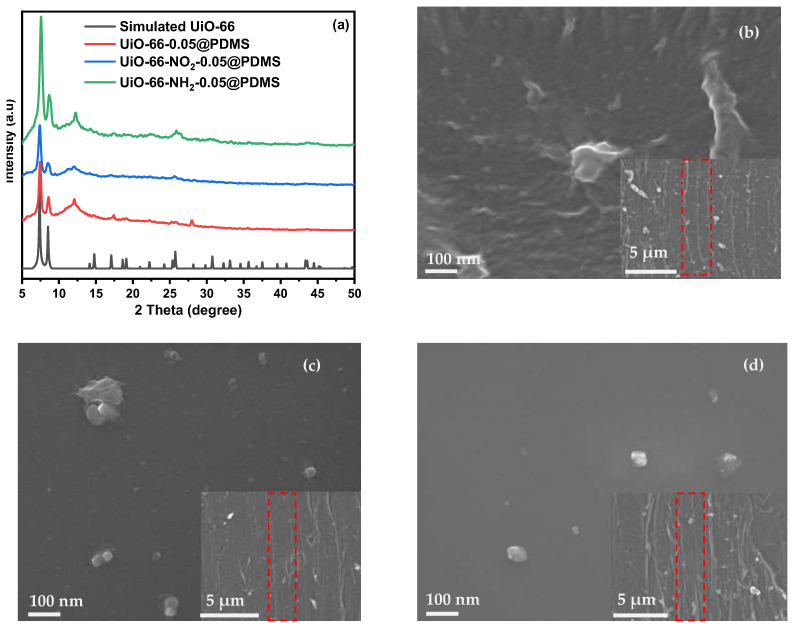
XRD patterns of different Zr-MOF@PDMS membranes (**a**) and SEM images of the membrane surface and cross-section morphologies (**b**–**d**).

The practical synthesis of three Zr-MOFs, namely UiO-66, UiO-66-NO_2_ and UiO-66-NH_2_, has been achieved using the procedures documented in the published literature [16]. The molecular structures of the three Zr-MOFs are shown in Figure S1a. The X-ray diffraction (XRD) patterns of the Zr-MOFs are depicted in Figure S1b. Figure S2 shows the defects in UiO-66 measurement by an acid–base titration and the defect calculations are exhibited in (Tables S1 and S2). The experimental patterns concur highly with those reported in the published literature [16,17,18]. SEM images in Figure S1c–e exhibit that these Zr-MOFs appear as irregular granules with different nano-sizes. After incorporating these Zr-MOFs into the PDMS matrix, the Zr-MOF-0.05@PDMS membranes have the XRD patterns with the prominent characteristic peaks of the three Zr-MOFs (Figure 1a), indicating that the original structures of MOFs are maintained in the PDMS matrix. The surface morphology and cross-section of the three Zr-MOF-0.05@PDMS membranes are characterized via SEM, as seen in Figure 1b–d. They show that the membranes are compact without apparent surface defects, and there is no severe agglomeration of MOFs in the membrane matrix. All the MOF crystals keep their original morphology when dispersed in the polymer matrix. The exceptional dispersion and contact between Zr-MOFs and PDMS can be attributed to the abundance of organic linkers present in the Zr-MOFs, the flexible nature of PDMS and the hydrophobic properties of the polymer chains [19,20]. The membranes were imaged using a Leica DM IL LED laboratory microscope with interchangeable S40/0.45 condenser lenses. The results, presented in Figure S3, confirmed a reduction in membrane defects following the incorporation of MOFs, aligning with the findings reported in the literature [21]. Additionally, the cross-sectional view of the membrane surface shows the Zr-MOFs inside the membrane nanochannels (red dotted box).

### 2.2. Optimization of the Zr-MOF@PDMS Membrane

**Figure 2:**
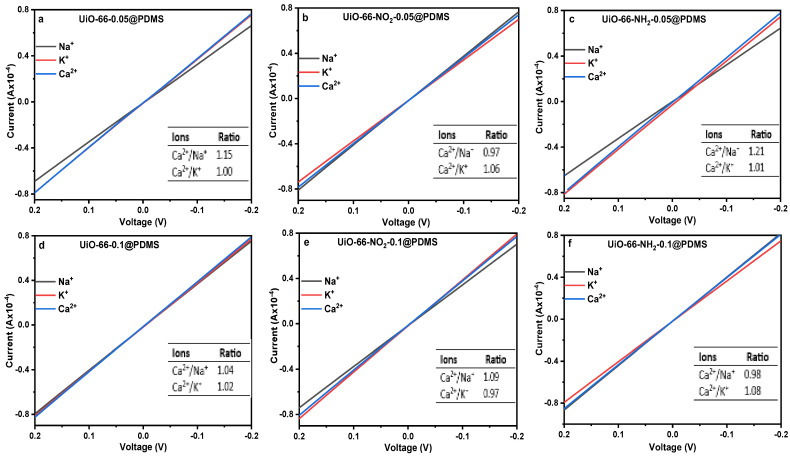
*I–V* curves of Zr-MOFs@PDMS membranes and the separation ratio (insert tables) (thickness: 600 ± 0.01 μm, pH = 7.42).

The content of Zr-MOF@PDMS membrane is considered for determining the optimum amount of doping Zr-MOFs with the PDMS matrix to obtain a high ion transport rate. The *I–V* curves of Zr-MOF@PDMS are shown in Figure 2. It can be seen from Table S3 that the difference in conductance values of ions in the Zr-MOF-0.05@PDMS membrane (Figure 2a–c) are more significant than that in the Zr-MOF-0.1@PDMS membrane (Figure 2d–f). It suggests that ions could transport through the membrane more efficiently by reducing ion transmembrane resistance in case of the lower content of Zr-MOFs (2.5 wt.%) because the higher content of Zr-MOFs over PDMS is more than (2.5 wt.%), which makes the membrane is too crisp to apply in the test. Hence, 2.5 wt.% is chosen as the optimal content of three Zr-MOFs over PDMS in our experiment at the constant thickness of 600 ± 0.01 μm. We confirmed the role of Zr-MOFs by changing the polymer matrix by utilizing PVDF as presented in Figure S4. The findings confirm that the pore channels provided by Zr-MOFs facilitate the separation of ions.

### 2.3. The Size-Sieving Effect

**Figure 3:**
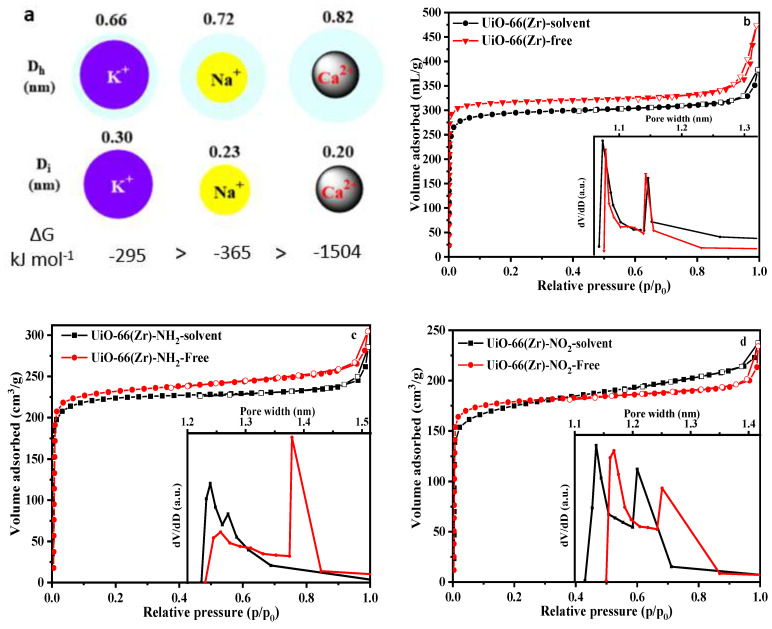
(**a**) Hydrated ionic diameter (*D*_h_), ionic diameter (*D*_i_), and hydration-free energy (*ΔG*) f different metal ions; (**b**–**d**) N_2_ sorption isotherm of different Zr-MOFs, and pore size distribution of Zr-MOFs (inset figures).

The *I–V* curves demonstrate the ion separation characteristics of the Zr-MOF-0.05@PDMS membrane, with the slope of the curve corresponding to the ion conductance values. A higher slope for a tested membrane, the ion transmembrane rate would have a higher velocity. The slope values observed in Figure 2 for Zr-MOF-0.05@PDMS membranes exhibit greater values than those observed in Figure S5 for pristine PDMS. The reason is that those Zr-MOFs possess many pores that serve as ion transport channels, decreasing ion transmembrane resistance [22]. Moreover, the tables in Figure 2 indicate that different Zr-MOF-0.05@PDMS membranes have varying ion separation effects, which may be affected by MOF pore sizes and ion diameters [23]. The hydrated ionic diameters of metal ions are demonstrated in Figure 3a [19,24], and Ca^2+^ < Na^+^ < K^+^ is the order of transport in the PDMS membrane (Figure S5). The order matches their ionic conductivity in an infinite dilution [25].

The porosity of UiO-66, UiO-66-NO_2_ and UiO-66-NH_2_ was examined by nitrogen adsorption analysis at 77 K, and their Brunauer–Emmett–Teller surface areas were 1115, 649 and 823 m^2^g^−1^, respectively (Figure 3). The pore size distributions of the Zr-MOFs, acquired from their N_2_ sorption isotherm curves, are presented in Figure 3b–d, which agree with the literature-reported pore diameters [16,26]. UiO-66-NH_2_ exhibits 1.24 nm pores (Figure 3c), bigger than any hydrated metal ions. Thus, metal ions and their hydration shells might cross the membrane. The order of transport rate for monovalent metal ions in UiO-66-NH_2_-0.05@PDMS is the same as that in PDMS. However, the separation ratio of Ca^2+^/Na^+^ and Ca^2+^/K^+^ is higher than that in PDMS. The reason may be that UiO-66-NH_2_ pores are Ca^2+^-restricted and the amino group interacted with Ca^2+^, which has the largest hydrated ionic diameter [27]. UiO-66-NO_2_ has a pore size of 1.13 nm (Figure 3d), higher than all the hydrated ionic diameters. K^+^ and Ca^2+^ were transported at the highest rate, but Na^+^ was transported at the slowest rate due to the affinity of the oxygen atom of the NO_2_ groups toward sodium ions, as will be elucidated by EDX mapping. UiO-66 exhibits 0.61 nm pores (Figure 3b), smaller than any hydrated ions. Hydrated metal ions will remove partial water molecules to enter MOFs for the shortest and least-resistance migratory path [23].

Consequently, metal ions with smaller ion diameters might shed portions of the water molecules in their hydration shells, allowing them to pass through the UiO-66-0.05@PDMS membrane more effortlessly. The separation ratio of the Ca^2+^/K^+^ and Ca^2+^/Na^+^ of UiO-66-0.05@PDMS membrane is slightly higher than that of PDMS and UiO-66-NO_2_-0.05@PDMS (Figure 2) due to the unhydrated ion diameters following the order Ca^2+^ < Na^+^ < K^+^. On the other hand, ions that have a lower hydration energy are more likely to leave their hydration shell [28]. Na^+^ has a lower hydration energy than Ca^2+^, suggesting that Na^+^ is more prone to losing its hydration shell than Ca^2+^. Then, a smaller dimension of unhydrated Na^+^ migrates faster than hydrated Ca^2+^.

Consequently, despite Ca^2+^ having a smaller unhydrated ion diameter than Na^+^, the UiO-66-0.05@PDMS membrane tends to Na^+^ migration, as the Ca^2+^/Na^+^ ratio exceeds 1. As a result, UiO-66-0.05@PDMS membrane pores could retain a greater quantity of hydrated Ca^2+^, resulting in its transport at the slowest but still lowest rate compared to UiO-66-NH_2_-0.05@PDMS due to the presence of NH_2_ groups in the UiO-66-NH_2_-0.05@PDMS membrane. MOF pore size generally affects ion selectivity, and tiny pores improve Na^+^ selectivity and Ca^2+^ trapping.

### 2.4. The Effect of Functional Groups on Zr-MOFs

**Figure 4:**
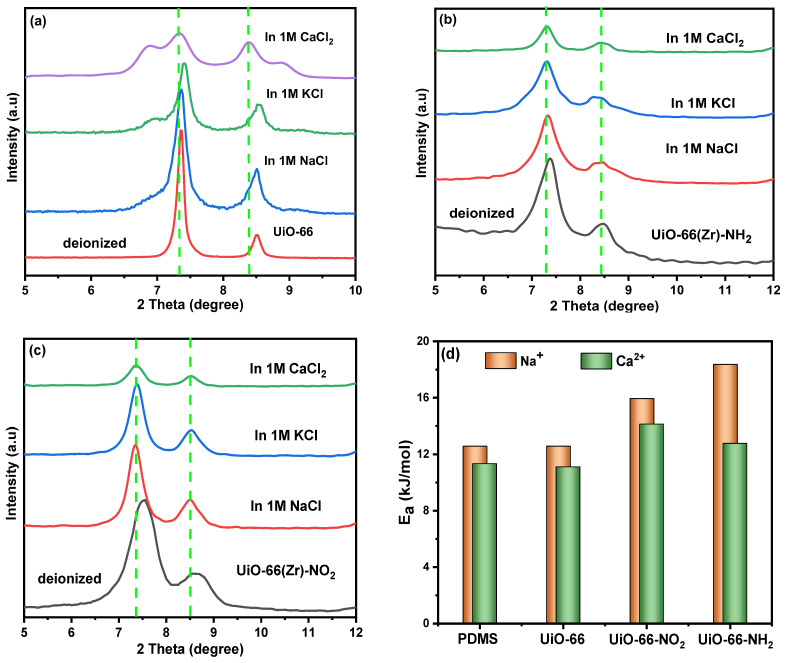
XRD patterns of different Zr-MOFs after immersing in deionized water, 1 mol L^−1^ NaCl, 1 mol L^−1^ KCl, and 1 mol L^−1^ CaCl_2_ solutions for 72 h, (**a**–**c**) and transport activation energies of Na^+^ and Ca^2+^ ions in Zr-MOF-0.05@PDMS and PDMS membranes (**d**).

As shown in Figure 3b,d, the pore size of UiO-66 is smaller than that of UiO-66-NH_2_ and UiO-66-NO_2_. However, the separation ratio of Ca^2+^/Na^+^ for the UiO-66-NH_2_-0.05-@PDMS membrane is still higher than that of the UiO-66-0.05@PDMS membrane. When only considering the size-sieving effect, the UiO-66-0.05@PDMS membrane should have higher selectivity than UiO-66-NH_2_-0.05@PDMS and UiO-66-NO_2_-0.05@PDMS. However, after decorating UiO-66 with amino groups, the slope values of UiO-66-NH_2_-0.05@PDMS from the *I–V* curve increased significantly compared with that of the two Zr-MOF membranes (Figure 2). To understand and compare the role of functional groups (-NH_2_ and -NO_2_) decorated with UiO-66, as seen in Figure 4, we investigated the Zr-MOFs’ affinity for the metal ions using XRD. After immersing in deionized water, 1 mol L^−1^ NaCl, 1 mol L^−1^ KCl and 1 mol L^−1^ CaCl_2_ solutions, the XRD peaks of the UiO-66 without functional groups were the same as before in both cases of Na^+^ and K^+^ ions. This result indicates there is little interaction between UiO-66 and these metal ions. However, the XRD patterns of UiO-66 in the case of Ca^2+^ show an apparent peak shift to a lower 2*θ* after immersing in 1 mol L^−1^ CaCl_2,_ and we observed that two peak shoulders at 2*θ* = 6.8° and 2*θ* = 8.9° confirm a strong interaction with Ca^2+^. Thus, Ca^2+^ diffusion rate enhancement in UiO-66 is lower than for other metal ions. However, in both cases of UiO-66-NO₂ and UiO-66-NH₂, the affinity is approximately the same. Due to the superior ion separation performance of UiO-66-NH₂-0.05@PDMS compared to the others, a membrane was prepared based on the results from the *I-V* curves. The EDX elemental analysis ensures the interaction between functional groups and ions in UiO-66-type materials. As shown in Figures S6 and S7, after immersing in 1 mol L^−1^ NaCl, 1 mol L^−1^ KCl and 1 mol L^−1^ CaCl_2_ solutions, the results confirmed that the trapping order of ions in the case of UiO-66 is Ca^2+^ > K^+^ > Na^+^; in the case of UiO-66-NO_2_, it is Na^+^ > K^+^ > Ca^2+^, and in case of UiO-66-NH_2_, it is Ca^2+^ > K^+^ > Na^+^. However, from Figure S6, the permeability of UiO-66 for Na^+^ is higher than other functionalized Zr-MOFs. Also, the trapping of UiO-66-NH_2_ for Ca^2+^ is higher than that of UiO-66 and UiO-66-NO_2_, as exhibited in Figure S7. This proves that the affinity of the amino groups for Ca^2+^ is much stronger than that of Na^+^ and K^+^ [29]. Additionally, to understand the role of functional groups and mechanisms, the ion transport activation energies (*Ε_a_*) through the UiO-66, UiO-66-NH_2_, UiO-66-NO_2_ and PDMS membranes were practically calculated according to the Arrhenius equation [30,31]:

(1) $$\ln\left(GT\right)=\ln\left(\beta \right)-\frac{E_{a}}{RT}$$

where *G* is the conductance of the ion across the membrane, *T* is the absolute temperature, *β* is the pre-exponential factor and *R* is the gas constant.

It is observed that unmodified PDMS and UiO-66 membranes exhibit no significant preference for cation selectivity. However, as the functional groups are altered from amino (-NH_2_) to nitro (-NO_2_), a notable change occurs in ion transport dynamics. Specifically, this modification results in a reduction in the energy activated required for calcium ion (Ca^2+^) transport, while simultaneously increasing the energy barrier for sodium ion (Na^+^) movement across the membrane (Figure 4d and Figure S8), in which the ion binding affinity of UiO-66-NO_2_ toward Na^+^ is higher than Ca^2+^. In contrast, the ion binding affinity of UiO-66-NH_2_ toward Ca^2+^ is higher than Na^+^. These findings confirm that Ca^2+^ interacts much more intensively with amino groups compared to nitro groups. Generally, the presence of amino groups in UiO-66-NH_2_ has the potential to enhance selectivity for Na^+^ over Ca^2+^, primarily due to the higher binding affinity of these groups for Ca^2+^. However, the increase in diffusion rate for Ca^2+^ is not as significant as it is for other metal ions.

### 2.5. The Stability of Zr-MOF-0.05@PDMS Membranes

**Figure 5:**
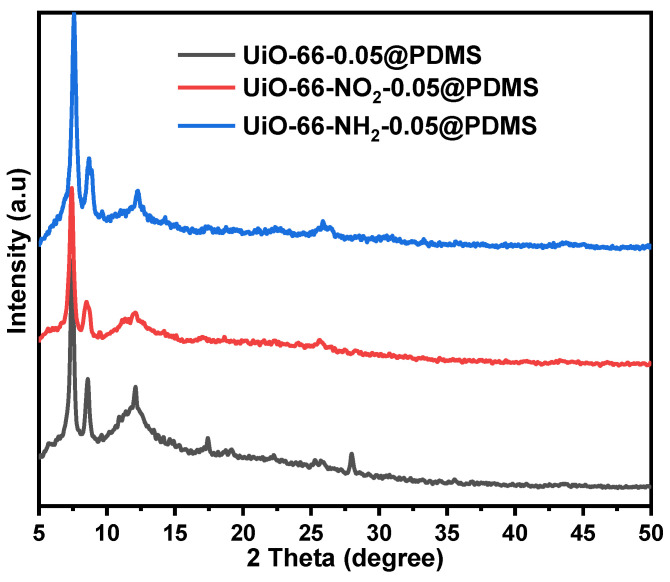
XRD patterns of the three Zr-MOF-0.05@PDMS after immersing in a saline solution for ten days.

This section assesses the stability of Zr-MOF-0.05@PDMS membranes. Figure 5 displays the X-ray diffraction (XRD) patterns of the three Zr-MOF-0.05@PDMS membranes. These membranes were immersed in a solution containing 1 mol L^−1^ NaCl and 1 mol L^−1^ CaCl_2_ at room temperature for varying durations. Three Zr-based MOFs—UiO-66, UiO-66-NO_2_ and UiO-66-NH_2_—maintained almost identical peaks in the PDMS matrix after 240 h of immersion in various saline solutions. The separation performance of the three Zr-MOF-0.05@PDMS membranes is tested using the (*I–V*) tests after ten days of immersion in a salt solution; the results are displayed in Figure S9 and Table S4. All the Zr-based MOF-0.05@PDMS membranes show the same separation performance as before (Table S4). The PDMS membrane and three Zr-based MOF-0.05@PDMS membranes are dimensionally stable in saline. After ten days in saline, they barely swelled or shrank. Overall, three Zr-based MOF-0.05@PDMS membranes were stable in test settings and can separate Na^+^ and Ca^2+^ from the solution.

### 2.6. Diffusion Experiment of Na^+^ and Ca^2+^

**Figure 6:**
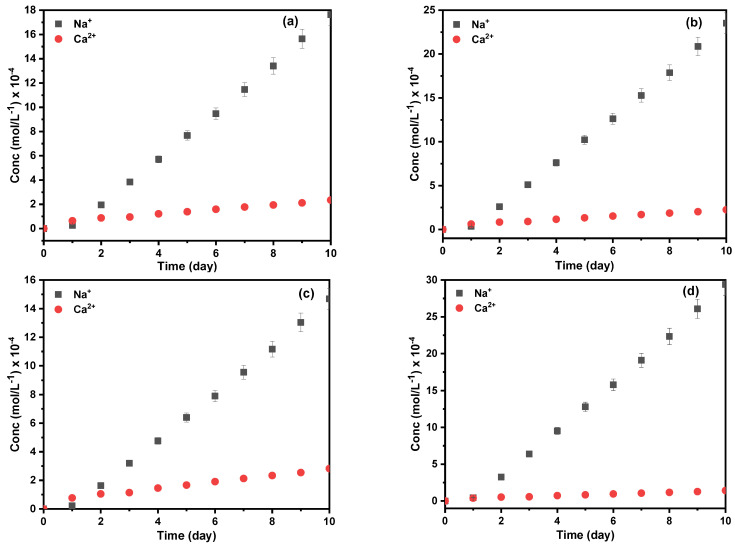
The concentration of Na^+^ and Ca^2+^ in the receiving solution at different times: (**a**) PDMS, (**b**) UiO-66-0.05@PDMS, (**c**) UiO-66-NO_2_-0.05@PDMS and (**d**) UiO-66-NH_2_-0.05@PDMS membranes.

**Figure 7:**
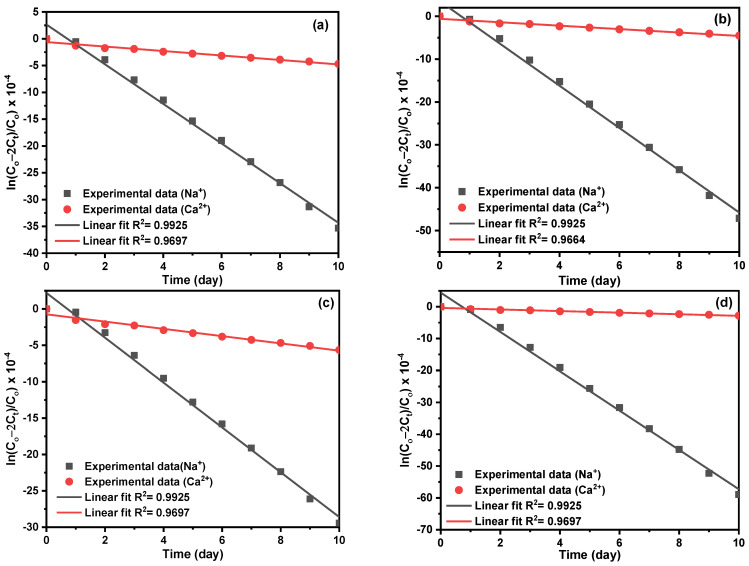
The plots of *ln* ((*C*_0_−2*C*_t_)/*C*_0_) vs. time. (feed solution: 1.0 mol L^−1^ NaCl and 1.0 mol L^−1^ CaCl_2_; receiving solution: deionized water, membrane: 600 ± 0.01 μm, (**a**) PDMS, (**b**) UiO-66-0.05@PDMS, (**c**) UiO-66-NO_2_-0.05@PDMS and (**d**) UiO-66-NH_2_-0.05@PDMS membranes; pH = 7.42).

The diffusion experiment of Na^+^ and Ca^2+^ compares the application potential of the optimized Zr-MOF-0.05@PDMS membranes with a thickness of 600 ± 0.01 μm. In the 10-day diffusion experiment (Figure 6), the concentration of Na^+^ and Ca^2+^ in the receiving solution increases with time, and the separation efficiency of Na^+^ and Ca^2+^ ions is aligned with the results obtained from the *I–V* curves. Further, the three Zr-MOF-0.05@PDMS membranes had the same structure as the pristine ones, indicating exceptional stability (Figure 7). In this section, ion diffusion coefficients were computed using diffusion data and ion conductivity from *I–V* curves. By using Fick’s law for the of the diffusion data, we can get the Na^+^ and Ca^2+^ diffusion coefficients, as shown in Equation (5). Combining Equations (6) and (7) with the *I–V* curves yield the diffusion coefficient; Figure 7 displays the findings. The slope of *ln*(*C*_0_−2*C_t_*)/*C*_0_) vs. time (*k*i), conductivity (*б*) and diffusion coefficient (*D*) are represented in Table S5. The similarity between the diffusion coefficients obtained from the *I–V* curves and the diffusion experiment suggests a high level of dependability in the accuracy of these measurements.

Table 1 provides empirical evidence that the permeation flow of Na^+^ in the PDMS membrane experiences a substantial increase following the incorporation of UiO-66-type materials. Moreover, a trade-off exists between the permeability and selectivity of the polydimethylsiloxane (PDMS) membrane. Specifically, a PDMS membrane with a satisfactory level of permeability has diminished Na^+^/Ca^2+^ selectivity (Table 1). After incorporating the Zr-MOFs, the PDMS-based hybrid membranes exhibit an enhanced Na^+^/Ca^2+^ separation performance. Particularly, the UiO-66-NH_2_-0.05@PDMS hybrid membrane exhibits a higher Na^+^ permeation flux and selectivity (*β*_Na+/Ca2+_) compared with the original PDMS membrane and other prepared hybrid membranes. The order of the permeation flux and selectivity (*β*_Na+/Ca2+_) are UiO-66-NH_2_ > UiO-66 > PDMS > UiO-66-NO_2_. The UiO-66-NH_2_-0.05@PDMS membrane achieves the highest Na^+^ permeation flux (2.33 × 10^−4^ mol·m^−2^·h^−1^) and selectivity (*β*_Na+/Ca2+_) = 11.2)). Also, we compared the transport rate and selectivity of Na^+^ and Ca^2+^ with those in published papers, as shown in Table 2. The selectivity of Na^+^ in UiO-66-NH_2_-0.05@PDMS was higher than that in ZIF-8/GO/AAO, UiO-66/PET, and smaller than the MOF membranes that contain sulfonated polyelectrolytes [32]. Additionally, the cation exchange capacity (CEC) for Na⁺ and Ca^2^⁺ ions was measured for both PDMS and UiO-66-NH_2_-0.05@PDMS, as detailed in the Supplementary Materials. The CEC values for UiO-66-NH_2_-0.05@PDMS were found to be 0.673 meq/g for Na⁺ and 1.353 meq/g for Ca^2^⁺, compared to 0.636 meq/g and 1.275 meq/g, respectively, for PDMS alone. These results indicate an enhanced cation exchange capacity in UiO-66-NH_2_-0.05@PDMS, particularly for Ca^2^⁺ ions. This enhancement may be attributed to the improved ion interaction sites provided by the incorporated UiO-66-NH_2_, suggesting its potential for applications requiring selective ion exchange.

Generally, the inclusion of Zr-MOFs in the PDMS-based hybrid membrane leads to enhancements in both selectivity and permeability. The enhanced efficiency of ionic permeation and selectivity seen in the UiO-66-NH_2_-0.05@PDMS hybrid membrane may be ascribed to the inclusion of the NH_2_-group functional group and the size-sieving effects facilitated by the embedded UiO-66-type materials. Hence, the ability to build several ion transport channels is made feasible by the porous nature of Zr-MOFs. The immobilized functional groups within the frameworks can provide sites for ion migration/or restriction and can also help create extra paths for cation separation.

## 3. Experimental

### 3.1. Materials

Zirconium oxychloride octahydrate (ZrOCl_2_∙8H_2_O) (Innochem, Pyeongtaek, Republic of Korea, 99.9%), 1,4-benzenedicarboxylic acid (BDC) (Innochem, 99%), 2-amino-1,4-benzenedicarboxylic acid (BDC-NH_2_) (Sinopharm, Beijing, China, 99%), 2-nitro-1,4-benzenedicarboxylic acid (BDC-NO_2_) (Sinopharm, 99%), PDMS, curing agent, heptane (Innochem, 99.9%)_,_ methanol (Innochem, 99.9%), ethanol (Innochem, 99.9%), N, N-dimethylformamide (DMF) (MACKLIN, Shanghai, China, 99.5%), sodium chloride (Innochem, 99.8%), potassium chloride (Innochem, 99.5%), calcium chloride (Innochem, 99.9%), standard solution of calcium and sodium ions and nitric acid were purchased from Sinopharm and Innochem companies. All the chemicals were used without further purification. Deionized water (>18 MΩ∙cm) was produced by the Milli-Q Water System (Millipore, Nanjing, China).

### 3.2. Synthesis of Materials

#### 3.2.1. Preparation of the Zr-MOF@PDMS Membrane

UiO-66(Zr) and its functionalized materials (UiO-66(Zr)-NH_2_, UiO-66(Zr)-NO_2_) were synthesized according to our published papers [6,16], and the preparation details are shown in the Supporting Information. Using these Zr-MOFs, we prepared Zr-MOF@PDMS membranes via a casting method.

**Figure 8:**
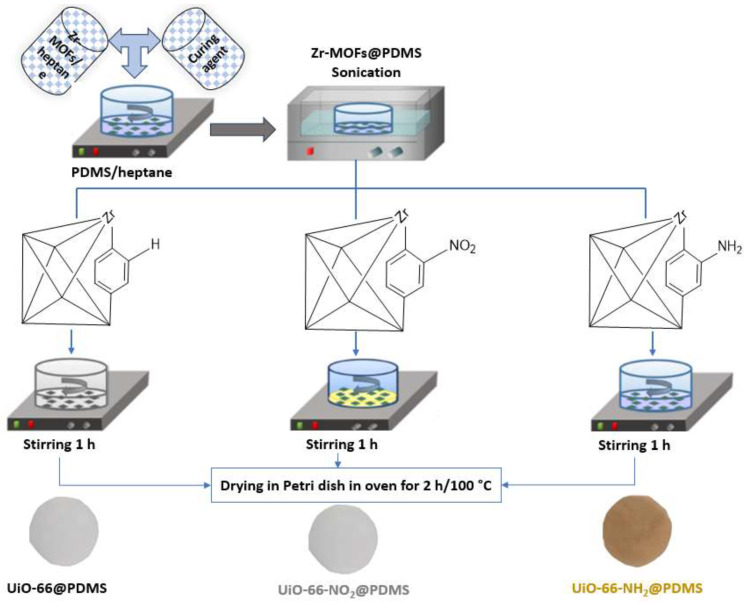
Schematic procedure for the synthesis of Zr-MOF@PDMS membranes.

Firstly, a solution was prepared by dissolving 2 g of PDMS in 2 g of heptane. Subsequently, Zr-MOF powder was dispersed in 1 mL of heptane and subjected to sonication for 10 min. The resulting dispersion was introduced into the above solution while stirred for one hour. The Zr-MOFs had a 2.5% or 5% weight ratio over PDMS. Subsequently, the homogeneous liquid was poured onto a petri dish of 5.5 × 5.5 cm and subjected to a temperature of 100 °C for 2 h, following a 5 min vacuum. Ultimately, the membrane was detached from the petri dish, and the thickness of the membrane was measured by a micrometer, which was 600 ± 0.01 μm. The membrane was named Zr-MOF-0.05@PDMS or Zr-MOF-0.1@PDMS according to the weight ratio of Zr-MOFs over PDMS added in the casting solution, as shown in Figure 8.

#### 3.2.2. Characterization

The surface and cross-sectional morphologies of Zr-MOFs and Zr-MOF@PDMS membranes were characterized via scanning electron microscopy (SEM, JSM-IT800, JEOL, Akishima, Japan). The structure of Zr-MOFs and Zr-MOF@PDMS membranes was detected by X-ray diffraction (XRD, D/Max-2500VPC, Rigaku, Tokyo, Japan) a scan rate of 10° per minute over a 2θ range of 5° to 70°. The N_2_ sorption isotherm of the three Zr-MOFs was recorded on BSD-PS1-1517 after degassing at 150 °C for 12 h. EDX mapping was carried out to determine the elemental composition of Zr-MOFs after immersing it in deionized water, 1 mol L^−1^ NaCl, 1 mol L^−1^ KCl and 1 mol L^−1^ CaCl_2_ for 72 h, and drying in the oven at 80 °C for 12 h. The electrochemical measurements were performed using a Potentisosat/Galvanostat instrument (CHI760E).

#### 3.2.3. Selective Ion Transport Properties

**Figure 9:**
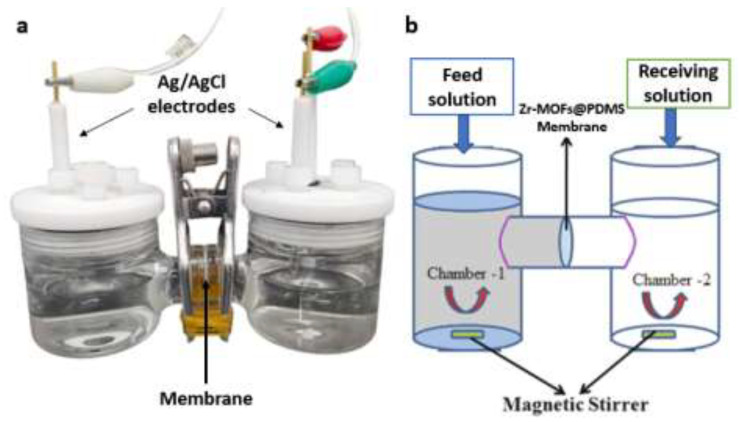
The photograph of the setup for ion conductivity (**a**) and schematic diagram of the test of ion separation (**b**).

The ion selection property was investigated by measuring the current–voltage (*I–V*) using the two-compartment transport cell. The *I–V* plots were conducted to assess and evaluate the ion transport characteristics of the Zr-MOF@PDMS membranes. The experimental procedures were conducted under ambient conditions within a two-compartment transport cell (Figure 9). A clamp was used to sandwich the Zr-MOF@PDMS membranes between the two compartments. The experiment involved the insertion of two Ag/AgCl electrodes into both the feed and receiving solution. A total volume of 50.0 mL of NaCl, KCl and CaCl_2_ at a concentration of 1.0 mol L^−1^ was introduced into both compartments. Before measurement, the Zr-MOF@PDMS membranes were submerged in a relevant solution for 12 h.

### 3.3. Ion Diffusion Experiment

The separation performance was evaluated using batch separation studies utilizing synthetic solutions containing sodium and calcium cations. The experimental procedure involved conducting an ion diffusion experiment within a two-compartment transport cell separated by a Zr-MOF-0.05@PDMS membrane with a thickness of 600 ± 0.01 μm. The feed solution for this experiment consists of a 50 mL mixture containing 1.0 (or 0.1) mol L^−1^ NaCl and 1.0 (or 0.1) mol L^−1^ CaCl_2_. The receiving solution, on the other hand, is 50 mL of deionized water. To mitigate the impact of concentration polarization on the permeation experiment, two compartments were subjected to stirring using a magnet at a velocity of 500 r/m. Periodic samples were collected from 0.5 mL aliquots of the receiving solution to assess the concentration of Ca^2+^ and Na^+^ ions using ICP-OES (Thermo Fisher iCAP 7000). The flux of the ion transport across membranes (*J*, mol·m^−2^·h^−1^) was calculated as follows:

(2) $$J_{Mn+}=\frac{V\cdot C_{t}}{A\cdot t}$$

where M^n+^ represents Na^+^ and Ca^2+^, *V* (50 mL) corresponds to the adequate volume of the receiving solution, *C_t_* (mol/L) is the molar concentration of the cation (M^n+^) in the receiving solutions at the sampling time *t* (day) and *A* (1.9022 cm^2^) is the membrane area.

The permeation experiments were conducted to estimate the hybrid membrane’s selectivity coefficient (*β*). The feed solution was a single kind of chloride solution with a 1.0 mol/L concentration. The chosen value was then derived using the following equation:

(3) $$\beta_{{Na}^{+}/{Ca}^{2+}}=\frac{J_{{Na}^{+}}}{J_{{Ca}^{2+}}}\frac{C_{{Ca}^{2+}}}{C_{{Na}^{+}}}$$

where *C_Na_^+^* and *C_Ca_^2+^* refer to the average concentrations of the Na^+^ and Ca^2+^ in the feed solutions, respectively.

The separation factor (*S.F.*) of the hybrid membranes was studied by permeation tests with mixed salt solutions as the feed solutions, and the value was calculated using the following equation:

(4) $${SF}_{N^{+}/{Ca}^{2+}}=\frac{C_{N^{+},P}/C_{{Ca}^{2+},p}}{C_{N^{+},f}/C_{{Ca2}^{+},f}}$$

where *C_N_^+^_,p_* is the sample concentration of the monovalent cations (Na^+^, and K^+^) in the receiving solution, *C*_Ca_^2+^_,p_ is the sample concentration of Ca^2+^ in the receiving solution, *C*_N_^+^_,f_ is the initial concentration of the monovalent cations in the feed solution and *C*_Ca_^2+^_,f_ is the initial concentration of Ca^2+^ in the feed solution.

The diffusion coefficient (D, cm^2^ s^−1^) could be calculated from the following Fick’s law [19,36]:

(5) $$ln\frac{C_{0}-{2C}_{t}}{C_{0}}=-\frac{2AD}{VL}t$$

where *C_t_* (mol L^−1^) and *C*_0_ (1.0 mol L^−1^) are the concentration of Ca^2+^ in the receiving solution at a selected time and feed solution at the initial time, respectively, A (1.9022 cm^2^) is the membrane area, *L* (cm) is the thickness of the membrane, *V* (50 mL) is the volume of the receiving solution and *t* (day) is the selected time of transport. Using the following equations, we also used the current–voltage (*I–V*) curves to estimate the diffusion coefficients of Na^+^, K^+^ and Ca^2+^ in the Zr-MOF@PDMS membranes. The ionic conductivity (σ, S cm^−1^) was calculated as follows [37]:

(6) $$\sigma =\frac{L}{RA}$$

*R* (Ω) is the resistance obtained from the slope of the *I–V* curves. The relationship of *σ* and *D* is expressed by the Nernst–Einstein (N.E.) relation [38]:

(7) $$\sigma =\frac{e^{2}N}{KT}D$$

where *e* (1.602 × 10^−19^ C) is electric charge, *N* (6.022 × 10^23^ mol^−1^) is Avogadro’s number, *k* (1.380 × 10^−23^ J K^−1^) is the Boltzmann constant, and *T* (298 K) is the temperature.

## 4. Conclusions

In summary, PDMS-based hybrid membranes incorporating Zr-MOFs have shown remarkable potential for selective Na^+^/Ca^2+^ separation. The integration of UiO-66, UiO-66-NO_2_ and UiO-66-NH_2_ MOFs into PDMS matrices enhanced selectivity and separation efficiency compared to pristine PDMS membranes. UiO-66-NH_2_-embedded membranes exhibited superior selectivity and achieved impressive separation efficiencies (64%) compared to pristine PDMS. The membranes demonstrated excellent stability and maintained their performance even after prolonged exposure to targeted cations, highlighting their robustness and potential for reuse. These findings offer exciting prospects for utilizing these hybrid membranes in various industrial and physiological applications requiring selective cation separation.

## Figures and Tables

**Table 1:** Comparison of the separation performance of different Zr-MOF-0.05@PDMS membranes.

Membrane	Separation Method	Na^+^ Transport Rate (mol·m^−2^·h^−1^)	Selectivity (Na^+^/Ca^2+^)	Separation Factor (Na^+^/Ca^2+^)
PDMS	Diffusion dialysis	1.4 × 10^−4^	4.03	4.02
UiO-66-0.05@PDMS	Diffusion dialysis	1.87 × 10^−4^	5.59	5.59
UiO-66-NO_2_-0.05@PDMS	Diffusion dialysis	1.17 × 10^−4^	2.80	2.79
UiO-66-NH_2_-0.05@PDMS	Diffusion dialysis	2.33 × 10^−4^	11.2	11.18

**Table 2:** Comparison of the ion selectivity of the UiO-66-NH_2_-0.05@PDMS membrane with other synthetic membranes.

Membrane	Target Ion	Separation Methods	Transport Rate (mol·m^−2^·h^−1^)	Selectivity	Ref.
HSO_3_-UiO-66@QPPO-20%	Li^+^Li^+^	Diffusion dialysis	6.750.238	Li^+^/Na^+^: 36Li^+^/Na^+^: 5.92	[32]
UiO-66-SO_3_H	Na^+^	Diffusion dialysis	2.80 × 10^−5^	Na^+^/Mg^2+^ > 140	[33]
UiO-66	Ca^2+^	Diffusion dialysis	0.778	Ca^2+^: 86.3%	[34]
ZIF-8/GO/AAO	Na^+^	electro dialysis	---	Na^+^/K^+^: 1.60	[35]
UiO-66/PET	Na^+^	electro dialysis	---	Na^+^/K^+^: 1.29	[35]
UiO-66-NH_2_-0.05@PDMS	Na^+^	Diffusion dialysis	2.33 × 10^−4^	Na^+^/Ca^2+^: 11.2	This work

## Data Availability

Data are contained within the article and Supplementary Materials.

## Supplementary Materials

The following supporting information can be downloaded at: https://www.mdpi.com/article/10.3390/molecules29225297/s1. Table Sx: The value of CEC for Na^+^ and Ca^2+^ over PDMS and UiO-66-NH2-0.05@PDMS membranes; Figure S1: The molecular structure of the Zr-MOFs (a), XRD patterns of Three Zr-MOFs samples (b), and SEM images of three Zr-MOFs (c–f); Figure S2: Acid-base titration curve and first derivative curve for UiO-66(Zr)-green; Table S1: Calculation of linker defects in UiO-66(Zr); Table S2: Calculation of missing linker and molecular formula of UiO-66(Zr); Figure S3: Microscopic images of membranes captured under a Leica DM IL LED microscope, illustrating the surface morphology and defect distribution. (a) PDMS, (b) UiO-66-0.05@PDMS, (c) UiO-66-NO_2_-0.05@PDMS, and (d) UiO-66-NH_2_-0.05@PDMS; Table S1: The conductance values of different ions in Zr-MOFs@PDMS membranes were studied by the current-voltage (*I–V*) measurement; Figure S4: *I–V* curves of Zr-MOFs@PVDF membranes (thickness: 600 ± 0.01 μm, pH = 7.42); Figure S5: *I–V* curves of PDMS membranes (thickness: 600 ± 0.01 μm, pH = 7.42); Figure S6: EDX-mapping of three Zr-MOFs after immersing in 1 mol L^−1^ NaCl, 1 mol L^−1^ KCl, and 1 mol L^−1^ CaCl_2_ solution for 72 h; Figure S7: Summary diagram for the EDX of Zr-MOFs in different saline solutions; Figure S8: *I–V* curves of UiO-66-NH_2_-0.05@PDMS and UiO-66-NO_2_-0.05@PDMS membranes (thickness: 600 ± 0.01 μm, pH= 7.42) at different temperatures (a–d), and the relation between ln(*GT*) against reciprocal of temperature (*1/T*). Figure S9: *I–V* curves of three Zr-MOFs-0.05@PDMS membranes after immersing in saline solution for 10 days (thickness: 600 ± 0.01 μm, pH = 7.42); Table S4: The slope and diffusion coefficient values of Na^+^ and Ca^2+^ ions in Zr-MOFs@PDMS membranes after immersing in saline solution for 10 days; Table S5: The slope of *ln((C_0_-2C_t_)/C_0_)* vs time (*k*i), conductivity (*б*), and diffusion coefficient (*D*) of Zr-MOFs-0.05@PDMS membranes (thickness: 600 ± 0.01 μm). Supporting information includes details about the preparation of the three Zr-MOFs, the slope from *I–V* curves, the EDX of Zr-MOFs after immersing in a saline solution for 72 h, and the *I–V* curve of membranes after immersing in saline solutions for ten days.
