# Supplementary material for: Engineering Ion Affinity of Zr-MOF Hybrid PDMS Membranes for the Selective Separation of Na+/Ca2+
Source: Molecules. 2024 Nov 9;29(22):5297. doi: 10.3390/molecules29225297 (PMC11596945; doi:10.3390/molecules29225297)
Supplement: Supplementary file 1 [file molecules-29-05297-s001.zip › molecules-3281035-supplementary.pdf]

Engineering Ion Affinity of Zr-MOFs Hybrid PDMS Membranes for Efficiently The Selective Separation of Na^+/Ca^{2+}

Ahmed S. Abou- Elyazed[1,2], Xiaolin Li[1*], Jing Meng[3*]

[1]Institute of Intelligent Manufacturing Technology, Shenzhen Polytechnic University, Shenzhen, 518055, P. R. China

[2]Chemistry Department, Faculty of Science, Menoufia University, Shebin EL-Kom, 32512, Egypt.

[3]School of Civil Engineering, Nantong Institute of Technology, Nantong, 226002, China.

Corresponding Author: *E-mail: lixiaolin0427@szpu.edu.cn; mengjign@sohu.com

1. Preparation of UiO-66(Zr) type materials

UiO-66(Zr)-structured materials including UiO-66(Zr) and NH_2- and NO_2-group functionalized UiO-66(Zr) were synthesized by green method; $ZrOCl_2 \cdot 8H_2O$ (1.5 mmol) as metal precursor and BDC or functionalized BDC (1.5 mmol) were pulverized together for about 10 min at room temperature. Then the pulverized materials were moved into an autoclave at 130 ºC for 12 h. After cooling to room temperature, the resulting white solid was washed with 70 ºC ethanol and dried for 12 h at 150 ºC under vacuum as stated in the previous work. The samples were denoted as UiO-66(Zr)-green, UiO-66(Zr)-NH_2-green and UiO-66(Zr)-NO_2-green, respectively. Comparatively, UiO-66(Zr)-solvent was synthesized according to the literature.

2. Cation exchange capacity calculations

Step-by-Step Calculation

- **Prepare the Solutions**:
  - Prepare solutions of NaCl and $CaCl_2$ at known concentrations. Measure the initial concentration of Na^+ and Ca^{2+} ions in each solution.

- **Pass the Solutions through the Membrane**:
  - Allow the solutions to pass through the membrane for a set period, ensuring that the membrane has enough time to reach equilibrium with the ions (10 days).
- **Measure the Ion Concentrations Post-Membrane**:
  - After the solution passes through the membrane, measure the concentration of Na^+ and Ca^{2+} ions on the permeate side (the side after the membrane) using ICP analysis.
- **Calculate the amount of Cations Adsorbed by the Membrane**:
  - Calculate the difference in ion concentration between the initial solution (feed compartment) and the permeate (receiving compartment). This difference represents the number of ions adsorbed by the membrane.
  - For example:

$$\Delta[Na^+] = [Na^+]_{initial} - [Na^+]_{permeate}$$

$$\Delta[Ca^{2+}] = [Ca^{2+}]_{initial} - [Ca^{2+}]_{permeate}$$

- **Convert to Milliequivalents**:
  - Since CEC is typically reported in milliequivalents, convert the adsorbed ion concentration to milliequivalents (meq).
  - For Na^+:

$$\text{Milliequivalents of } Na^+ = \Delta[Na^+] \times \text{volume of solution (L) solution}$$

  - For Ca^{2+}: (which has a valency of 2):

$$\text{Milliequivalents of } Ca^{2+} = 2 \times \Delta[Ca^{2+}] \times \text{volume of solution (L) solution}$$

- **Normalize by the Dry Mass of the Membrane**:
  - The Weight of the dry membrane before the experiment (0.0739 g). Calculate the CEC by dividing the total milliequivalents of adsorbed cations by the mass of the dry membrane. The total volume of solution 50 mL.

$$\text{CEC (meq/g)} = \frac{\text{Total meq of adsorbed cations}}{\text{mass of dry membrane (g)}}$$

Calculation

The initial concentration of Na^+ and Ca^{2+} in the feeding solution equal 1 mol/L and the concentration of Na^+ and Ca^{2+} after diffusion for 10 days in the receiving solution equal 0.002935 mol/L for Na^+ and Ca^{2+} equal 0.0001544 mol/L through UiO-66-NH_2-0.05@PDMS membrane. Hence,

  - **For Na^+:**

$$\Delta[Na^+] = [Na^+]_{initial} - [Na^+]_{permeate} = 1 - 0.002935 = 0.997065 \text{ mol/L}$$

$$\text{Milliequivalents of } Na^+ = \Delta[Na^+] \times \text{volume of solution (L) solution}$$

$$= 0.997065 \text{ mol/L} \times 0.05 = 0.0498 \text{ meq/L}$$

$$\text{CEC (meq/g)} = \frac{\text{Total meq of adsorbed cations}}{\text{mass of dry membrane (g)}}$$

$$\text{CEC}\left(\frac{\text{meq}}{\text{g}}\right) = \frac{0.0498\,\frac{\text{meq}}{\text{L}}}{0.0739} = 0.673\,\frac{meq}{L.g}$$

- **For Ca^{2+}: (which has a valency of 2):**

$$\Delta[Ca^{2+}] = [Ca^{2+}]_{initial} - [Ca^{2+}]_{permeate} = 1 - 0.0001544 = 0.99984 \text{ mol/L}$$

Milliequivalents of $Ca^{2+} = 2 \times \Delta[Ca^{2+}] \times$ volume of solution (L) solution

$$= 2 \times 0.99984 \text{ mol/L} \times 0.05 = 0.09998 \text{ meq/L}$$

$$\text{CEC (meq/g)} = \frac{\text{Total meq of adsorbed cations}}{\text{mass of dry membrane (g)}}$$

$$\text{CEC}\left(\frac{\text{meq}}{\text{g}}\right) = \frac{0.09998\,\frac{\text{meq}}{\text{L}}}{0.0739} = 1.353\,\frac{meq}{L.g}$$

Table Sx. The value of CEC for Na^+ and Ca^{2+} over PDMS and UiO-66-NH_2-0.05@PDMS membranes.

**Membrane types**	**Cation exchange capacity (CEC)**	
	**For Na^+**	**For Ca^{2+}**
PDMS membrane [a]	0.636	1.275
UiO-66-NH_2-0.05@PDMS	0.673	1.353

[a]The weight of PDMS as dry membrane = 0.0784 g

3. Potentiometric acid-base titrations

Potentiometric acid-base titrations were completed with a metrohm titrando 905 autotitrator equipped with dosino 800, 20 mL and 10 mL dosing units using a procedure previously reported. 1-4 Prior to each titration, calibrations were performed with 2.00 and 9.00 metrohm buffer solutions. Sample preparation entailed using approximately 50 mg of sample (previously activated for 12 h at 150 °C under the condition of vacuum) that were crushed into a fine powder with a plastic spatula in a 100 mL beaker. Then approximately 50 mL of 0.01 M $NaNO_3$ solution was added and allowed to equilibrate for 18 h. Preceding each titration, a stir bar was added to the beaker and the *pH* was adjusted to a value of 3.00 with 0.1 M HCl. Following, the solution was titrated with a 0.1 M NaOH solution to a pH value of 10.5-11.0 with an injection volume of 0.025 mL at a rate of 0.020 mL/min. To better visualize the equivalence points, the first derivative of the titration curve (*pH* vs. Volume of 0.1 M NaOH Added) was taken (*dpH/dV* vs. Volume of 0.1 M NaOH added). The first derivative plots were then subjected to

lorentzian function curve-fitting with origin proV8.5 multiple peak fitting function. *pKa* values were determined by first identifying the equivalence volumes on the first derivative plots and taking the pH at one-half of the titrant volume added to reach the equivalence point.

Explanation of calculations: Because titrations are quantitative in nature and that defect sites inherently have titratable protons (under our conditions with a solution pH of 3), we are able to determine the number of defect sites in each sample. To determine the number of Zr-OH_2, Zr-OH that are present, we simply calculate the amount of NaOH titrant consumed for each species. With respect to the Zr-OH_2 species, we take the difference in the amount of NaOH titrant consumed between the second (Zr-OH_2) and first (μ_3-OH) equivalence points (each terminal water is deprotonated once, yielding an additional terminal hydroxyl group which we assume to be equivalent to the terminal hydroxyl group that was already present in the pair). In a similar fashion, the number of Zr-OH species that are present can be found by taking the difference in NaOH titrant consumed between the third (Zr-OH) and second (Zr-OH_2) equivalence points and removal yielding an additional terminal hydroxyl group. The relative amounts of defect sites from Zr-OH_2 and Zr-OH, with respect to each other, are then calculated and assigned to the variables y and z, respectively. These values are then related to the molecular formula of UiO-66 that incorporates Zr-OH_2 and Zr-OH compensating missing linker defects, $Zr_6O_4(OH)_4(bdc)_{6-x}[(OH_2)_y(OH)_z]_4$, where x is the number of missing linkers in an ideal $Zr_6O_4(OH)_4(bdc)_6$ cluster. Following, the number of missing linkers can be realized by comparing the experimental value of titrated protons from the calculated theoretical values with different numbers of missing linkers.

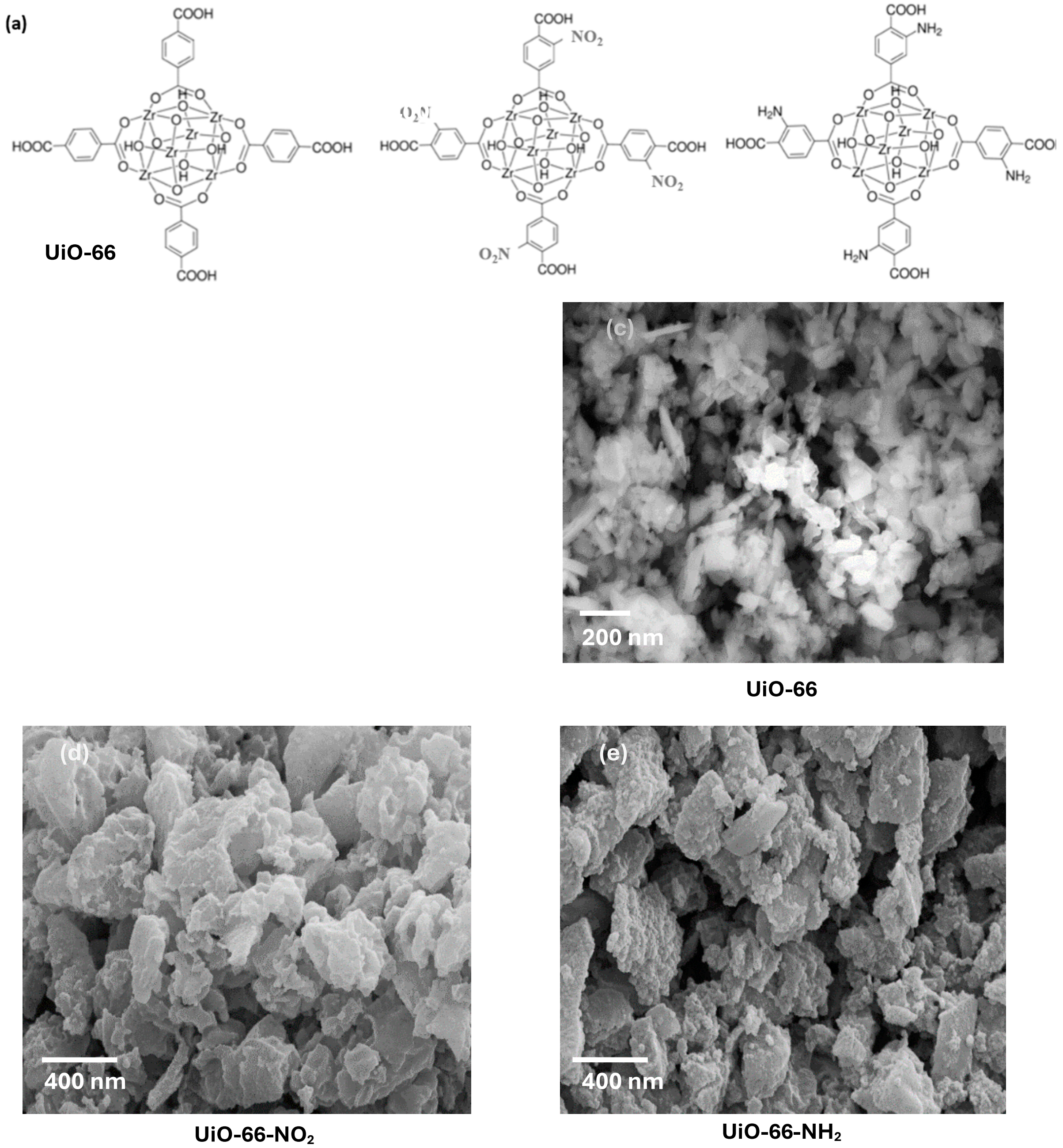

Figure S1. The molecular structure of the Zr-MOFs (a), XRD patterns of Three Zr-MOFs samples (b), and SEM images of three Zr-MOFs (c-f).

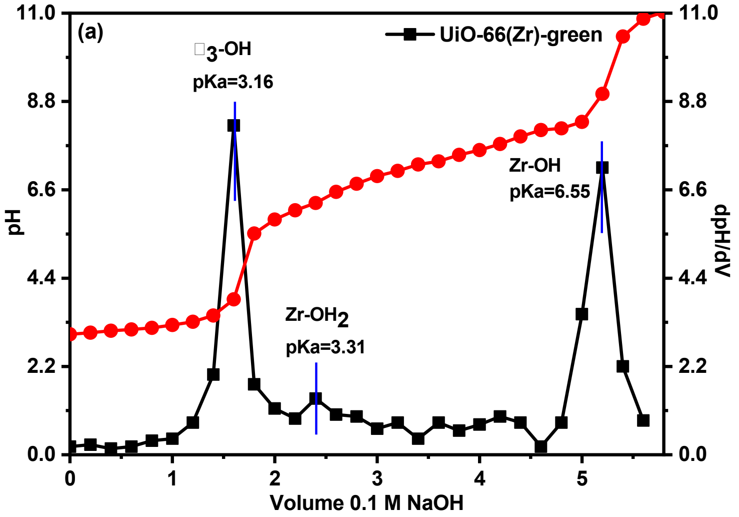

Figure S2. Acid-base titration curve and first derivative curve for UiO-66(Zr)-green.

Table S1. Calculation of linker defects in UiO-66(Zr).

**μ_3-OH**		
	1.6 mL	mL NaOH
	0.16	mmol protons from μ_3-OH
**Zr-OH_2**		
EP2-EP1	2.406-1.6 = 0.806 mL	mL NaOH
	0.0806	mmol protons from Zr-OH_2
**Zr-OH**		
EP3-EP2	5.197-2.406 = 2.791 mL	mL NaOH
	0.2791	mmol protons from Zr-OH
	**0.3597**	Total mmol defect sites
Relative amount of defect sites from Zr-OH_2=		22.4%
Relative amount of defect sites from Zr-OH=		77.6%

Table S2. Calculation of missing linker and molecular formula of UiO-66(Zr).

Missing linker	Molecular formula	Mw	mmoles H^+ from defects in 0.05 g sample
0	$Zr_6O_4(OH)_4(C_8H_4O_4)_6$	1664.06	0
1	$Zr_6O_4(OH)_4(C_8H_4O_4)_5[(H_2O)_{0.224}(OH)_{0.776}]_4$	1568.86	0.1275
1.5	$Zr_6O_4(OH)_4(C_8H_4O_4)_{4.5}[(H_2O)_{0.224}(OH)_{0.776}]_6$	1521.268	0.1972
1.53	$Zr_6O_4(OH)_4(C_8H_4O_4)_{4.47}[(H_2O)_{0.224}(OH)_{0.776}]_{6.12}$	1518.412	0.2015
1.8	$Zr_6O_4(OH)_4(C_8H_4O_4)_{4.2}[(H_2O)_{0.224}(OH)_{0.776}]_{7.2}$	1492.712	0.2412
2	$Zr_6O_4(OH)_4(C_8H_4O_4)_4[(H_2O)_{0.224}(OH)_{0.776}]_8$	1473.675	0.2714
2.5	$Zr_6O_4(OH)_4(C_8H_4O_4)_{3.5}[(H_2O)_{0.224}(OH)_{0.776}]_{10}$	1426.083	0.3506
**2.55**	$\mathbf{Zr_6O_4(OH)_4(C_8H_4O_4)_{3.444}[(H_2O)_{0.224}(OH)_{0.776}]_{10.224}}$	**1420.752**	**0.3597**
2.57	$Zr_6O_4(OH)_4(C_8H_4O_4)_{3.43}[(H_2O)_{0.224}(OH)_{0.776}]_{10.28}$	1419.42	0.3621
2.6	$Zr_6O_4(OH)_4(C_8H_4O_4)_{3.4}[(H_2O)_{0.224}(OH)_{0.776}]_{10.4}$	1416.564	0.3671
3	$Zr_6O_4(OH)_4(C_8H_4O_4)_3[(H_2O)_{0.224}(OH)_{0.776}]_{12}$	1378.49	0.4352

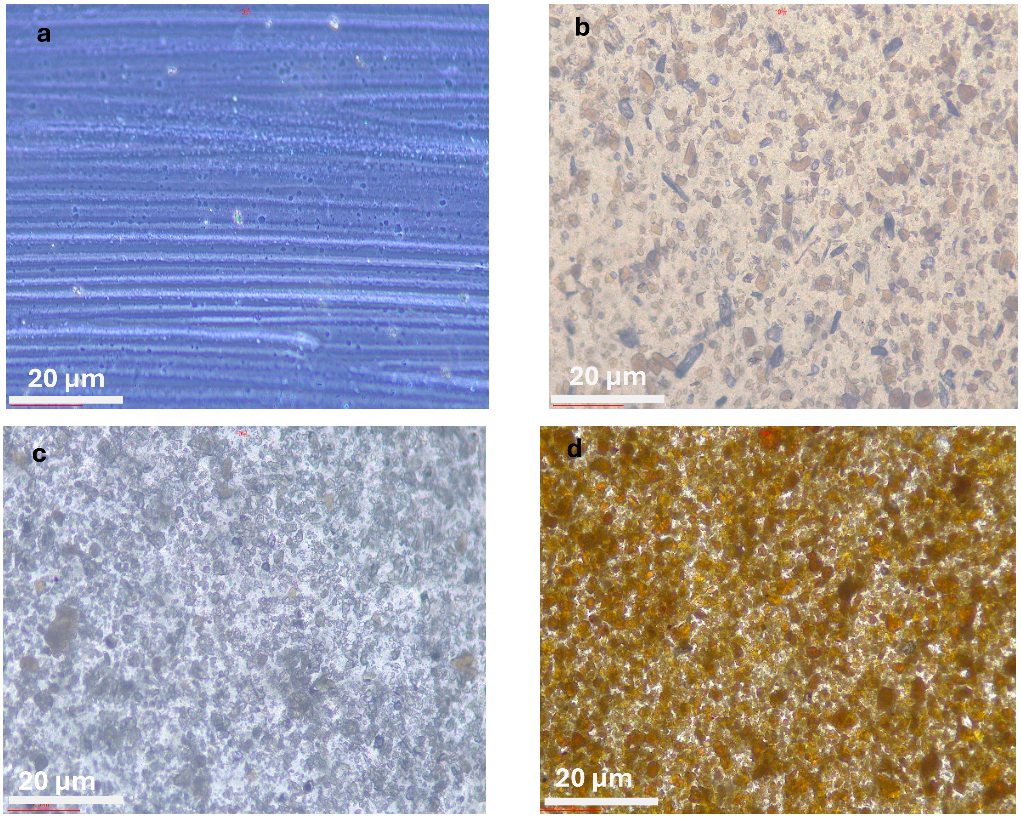

Figure S3. Microscopic images of membranes captured under a Leica DM IL LED microscope, illustrating the surface morphology and defect distribution. (a) PDMS, (b) UiO-66-0.05@PDMS, (c) UiO-66-NO_2-0.05@PDMS, and (d) UiO-66-NH_2-0.05@PDMS.

Table S1. The conductance values of different ions in Zr-MOFs@PDMS membranes were studied by the current-voltage (*I–V*) measurement.

Membrane	Na^+	K^+	Ca^{2+}	Ratio $Ca^{2+}/Na+$	Ratio Ca^{2+}/K^+
PDMS	3.80E-04	4.29E-04	4.20E-04	1.11	0.97
UiO-66-0.05@PDMS	3.37E-04	3.84E-04	3.87E-04	1.15	1.00
UiO-66-NH_2-0.05@PDMS	3.24E-04	3.89E-04	3.80E-04	1.21	1.01
UiO-66-NO_2-0.05@PDMS	3.93E-04	3.59E-04	3.80E-04	0.97	1.06
UiO-66-0.1@PDMS	3.86E-04	3.95E-04	4.03E-04	1.04	1.02
UiO-66-NH_2-0.1@PDMS	4.22E-04	3.85E-04	4.16E-04	0.98	1.08
UiO-66-NO_2-0.1@PDMS	3.60E-04	4.07E-04	3.94E-04	1.09	0.97

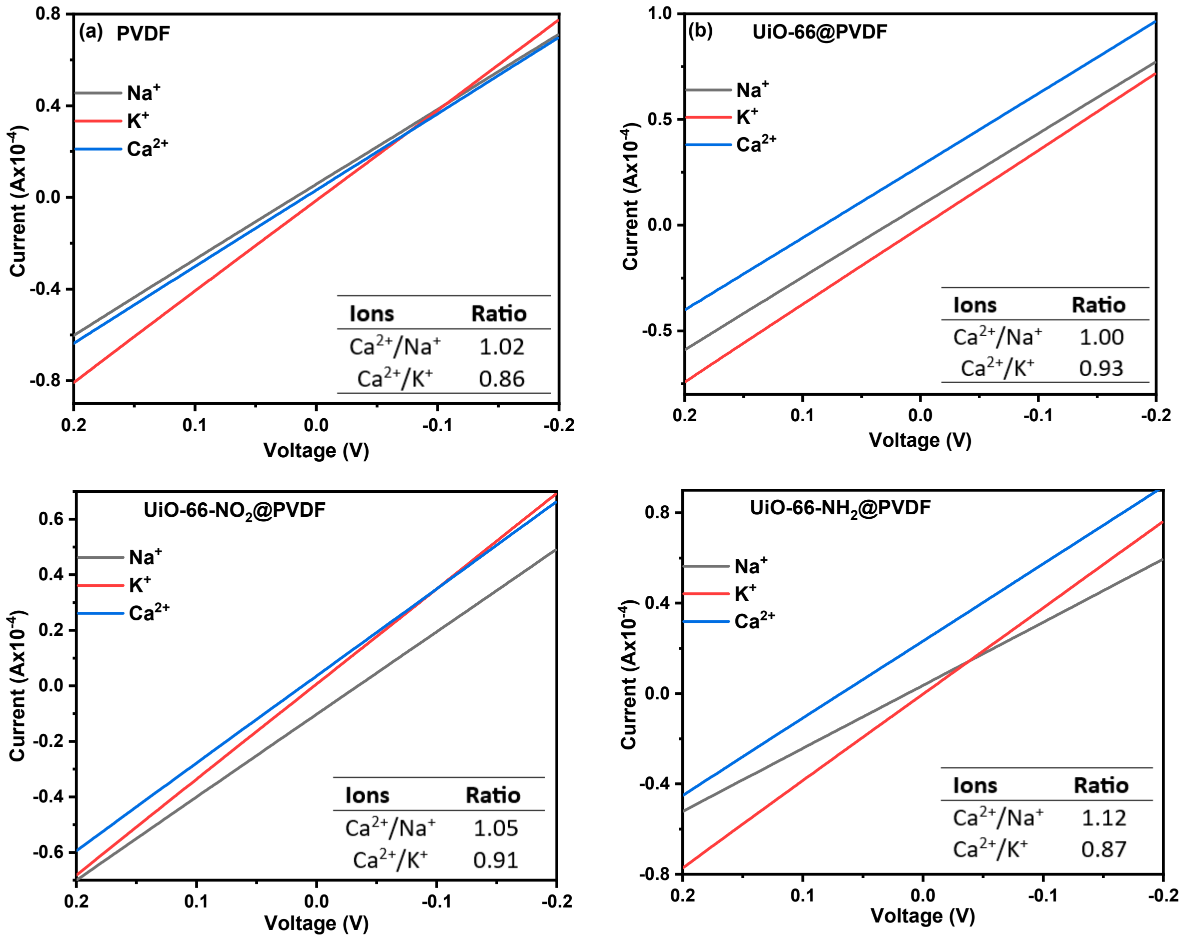

Figure S4. *I-V* curves of Zr-MOFs@PVDF membranes (thickness: 600 ±0.01 μm, pH= 7.42)

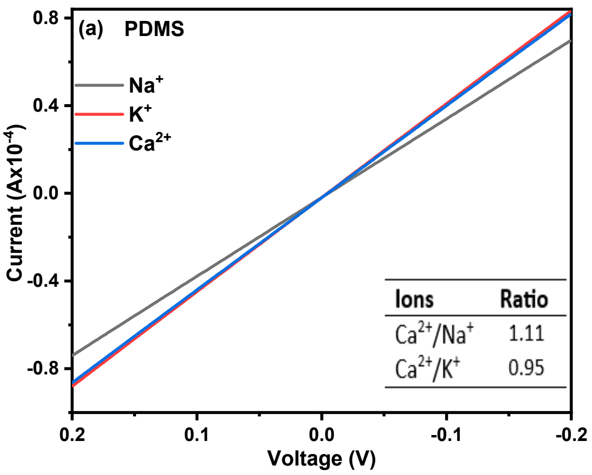

Figure S5. *I-V* curves of PDMS membranes (thickness: 600 ±0.01 μm, pH= 7.42).

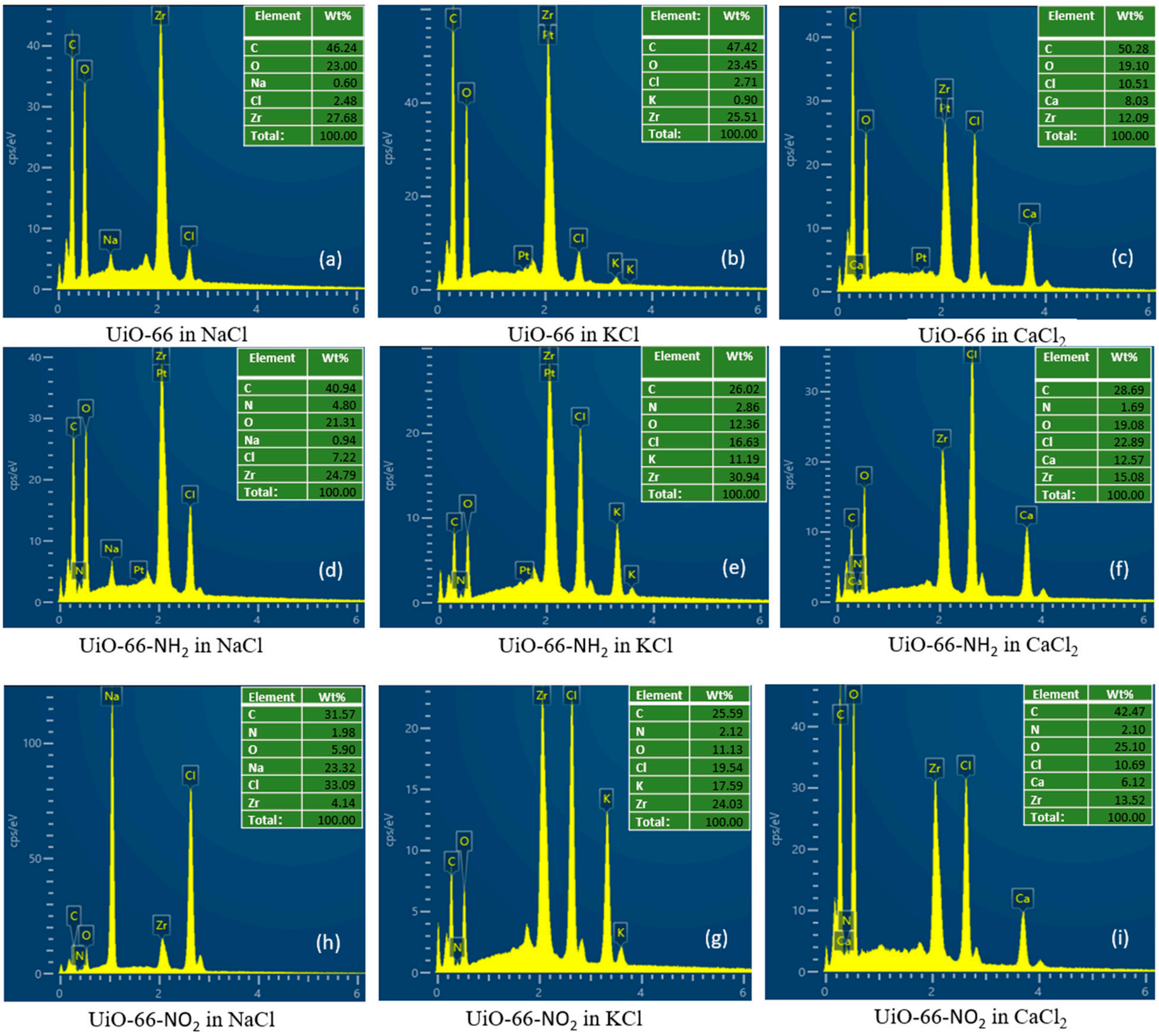

Figure S6. EDX-mapping of three Zr-MOFs after immersing in 1 mol L^{-1} NaCl, 1 mol L-1 KCl, and 1 mol L^{-1} $CaCl_2$ solution for 72 h.

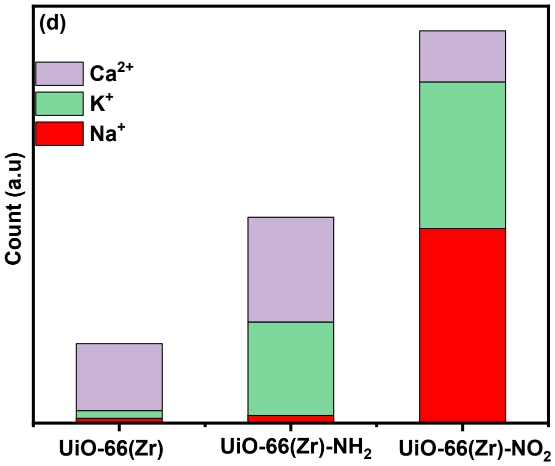

Figure S7. Summary diagram for the EDX of Zr-MOFs in different saline solutions.

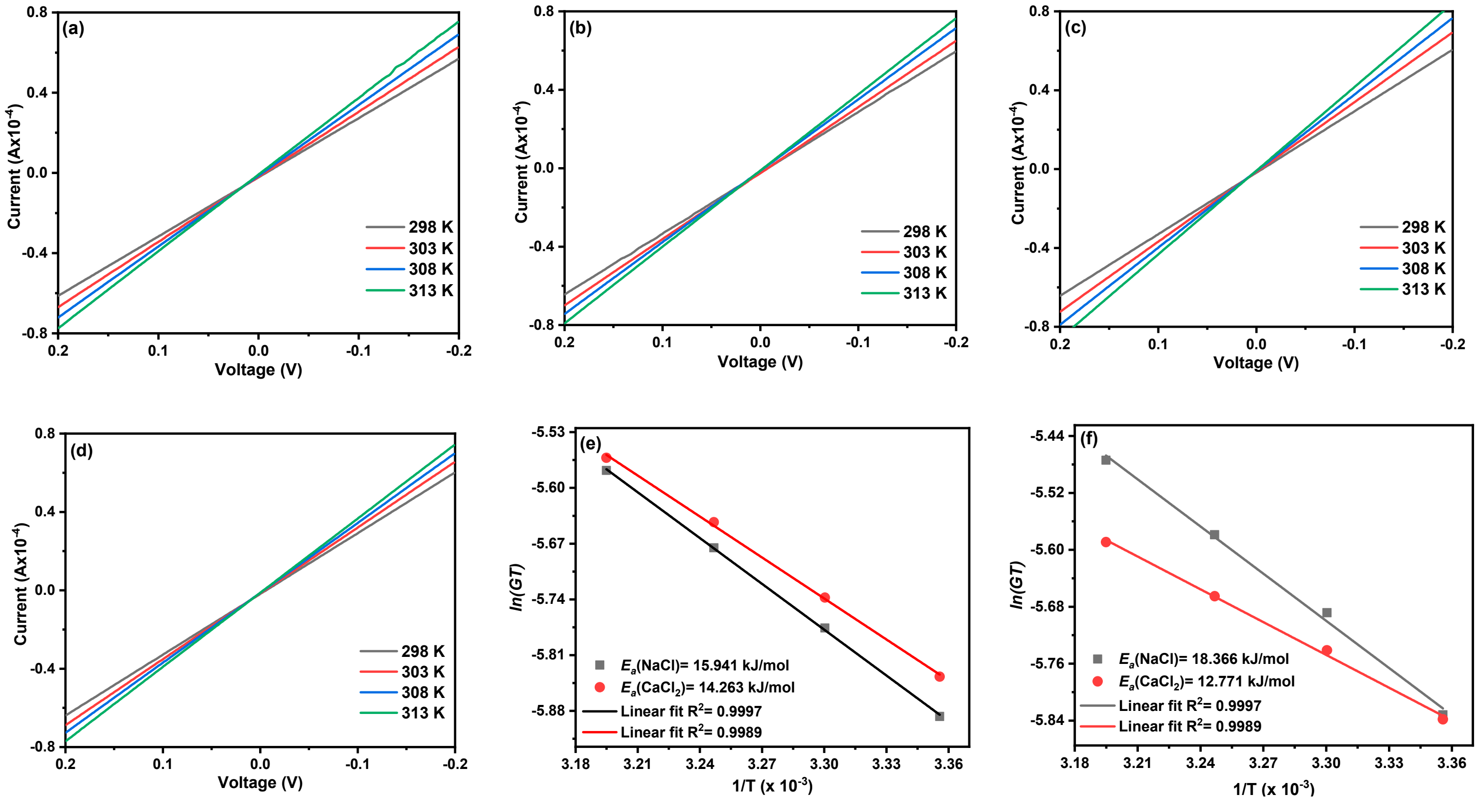

Figure S8. *I-V* curves of UiO-66-NH_2-0.05@PDMS and UiO-66-NO_2-0.05@PDMS membranes (thickness: 600 ±0.01 µm, pH= 7.42) at different temperatures (a-d), and the relation between ln(GT) against reciprocal of temperature (1/T).

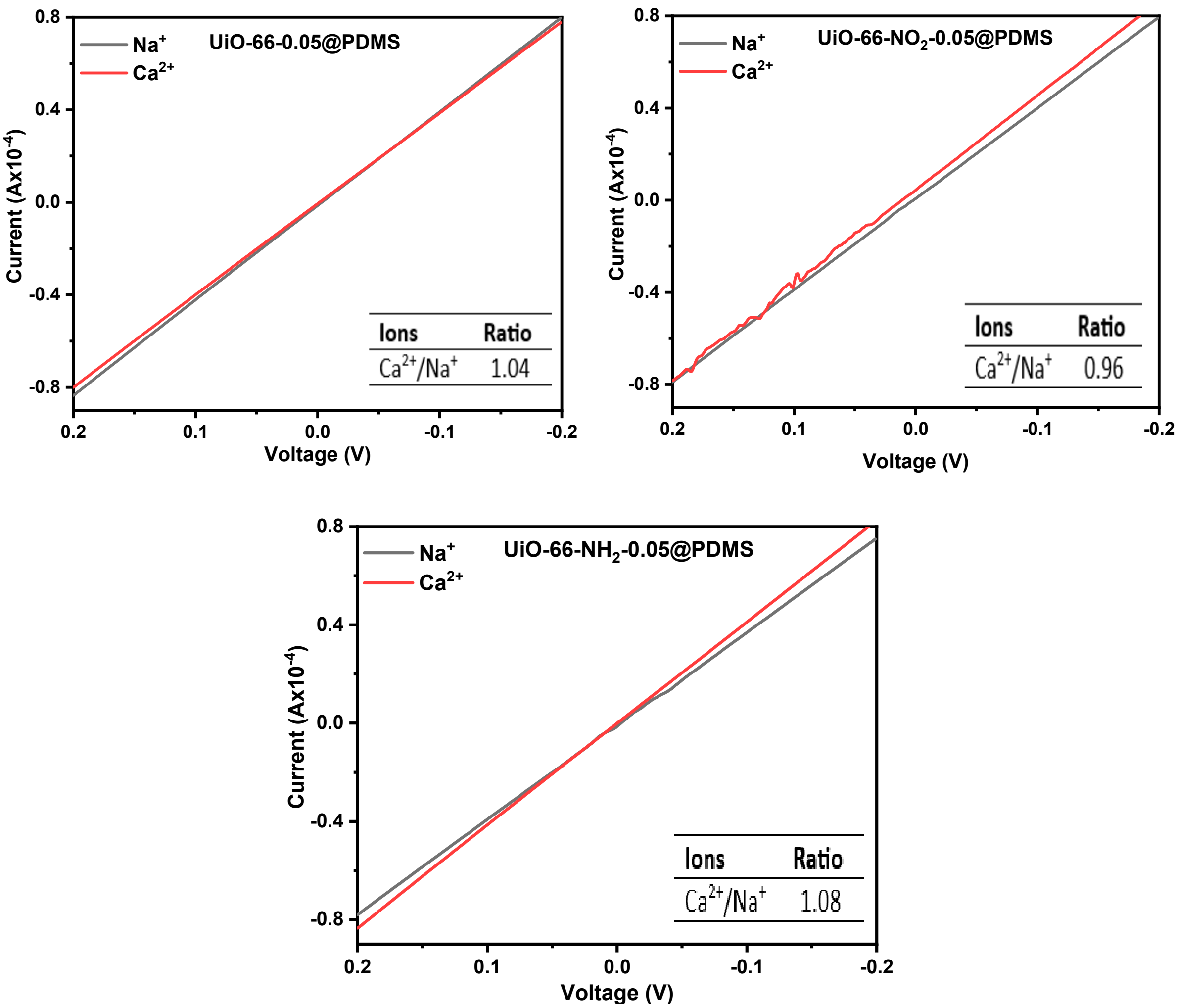

Figure S9. *I-V* curves of three Zr-MOFs-0.05@PDMS membranes after immersing in saline solution for 10 days (thickness: 600 ±0.01 μm, pH= 7.42).

Table S4. The slope and diffusion coefficient values of Na^+ and Ca^{2+} ions in Zr-MOFs@PDMS membranes after immersing in saline solution for 10 days.

**Membrane**	***Slope***		***D*** **($cm^2\ s^{-1}$)**		***Ratio*** ***Ca^{2+}/Na^+***
	Na^+	Ca^{2+}	Na^+	Ca^{2+}	
UiO-66-0.05@PDMS	4.08E-04	3.94E-04	3.42E-12	3.31E-12	1.04
UiO-66-NO_2-0.05@PDMS	3.95E-04	4.10E-04	3.32E-12	3.44E-12	0.96
UiO-66-NH_2-0.05@PDMS	3.82E-04	4.14E-04	3.21E-12	3.48E-12	1.08

Table S5. The slope of *ln((C₀-2Cₜ)/C₀)* vs time (*k*i), conductivity (*σ*), and diffusion coefficient (*D*) of Zr-MOFs-0.05@PDMS membranes (thickness: 600 ±0.01 μm).

Membrane	Ions	Diffusion		*I-V* curves	
		k_i	**D (cm^2 s^{-1})**	**σ (cm^{-1} S)**	**D (cm^2 s^{-1})**
PDMS	Na^+	-3.70E-05	3.36E-10	1.20E-05	3.19E-12
	Ca^{2+}	-4.16E-05	3.79E-10	1.32E-05	3.53E-12
UiO-66-0.05@PDMS	Na^+	-3.51E-05	3.18E-10	1.06E-05	2.83E-12
	Ca^{2+}	-4.00E-05	3.64E-10	1.22E-05	3.25E-12
UiO-66-NO_2-0.05@PDMS	Na^+	-5.12E-05	4.79E-10	1.24E-05	3.30E-12
	Ca^{2+}	-5.00E-05	4.56E-10	1.20E-05	3.19E-12
UiO-66-NH_2-0.05@PDMS	Na^+	-2.06E-05	1.89E-10	1.02E-05	2.62E-12
	Ca^{2+}	-2.50E-05	2.28E-10	1.20E-05	3.19E-12
